# Supplementary material for: Childhood trauma, PTSD/CPTSD and chronic pain: A systematic review
Source: PLoS One. 2024 Aug 30;19(8):e0309332. doi: 10.1371/journal.pone.0309332 (PMC11364226; doi:10.1371/journal.pone.0309332)
Supplement: Attachment — Submitted filename: Response To Reviewers.docx [file pone.0309332.s003.docx]

**Point-by-Point Rebuttal Letter**

Journal requirements

1. Please ensure that your manuscript meets PLOS ONE's style requirements, including those for file naming. The PLOS ONE style templates can be found at

<https://journals.plos.org/plosone/s/file?id=wjVg/PLOSOne_formatting_sample_main_body.pdf> and

<https://journals.plos.org/plosone/s/file?id=ba62/PLOSOne_formatting_sample_title_authors_affiliations.pdf>

**Answer: *We have checked the templates and made a few adjustments to meet the journal requirements, in particular with respect to headlines, Table titles, as well as file naming.***

2. Thank you for stating the following financial disclosure:

“No external funding existed for the study. But we acknowledge financial publication support by Centre Hospitalier Agen-Nérac. The funders had no role in study design, data collection and analysis, decision to publish, or preparation of the manuscript.”

At this time, please address the following queries:

a) Please clarify the sources of funding (financial or material support) for your study. List the grants or organizations that supported your study, including funding received from your institution.

**Answer:** ***None of the authors received material or financial funding for this study. However, the Centre Hospitalier Agen-Nérac agreed to pay the publication fee related to this article in PLOS ONE.***

b) State what role the funders took in the study. If the funders had no role in your study, please state: “The funders had no role in study design, data collection and analysis, decision to publish, or preparation of the manuscript.”

**Answer:** ***Apart from the above-mentioned publication fee, there were no funders involved in the study project.***

c) If any authors received a salary from any of your funders, please state which authors and which funders.

**Answer:** ***None of the authors received salary from funders to conduct this research.***

***The first author, Maria Karimov-Zwienenberg, is employee of the Centre Hospitalier Agen-Nérac and receives salary in the context of her clinical job which is unrelated to this research.***

d) If you did not receive any funding for this study, please state: “The authors received no specific funding for this work.”

**Answer:** ***“The authors received no specific funding for this work.”***

Please include your amended statements within your cover letter; we will change the online submission form on your behalf.

**Answer: *The following statement has been added to the cover letter (See file: Cover Letter_Updated Statement):***

***“The authors received no specific funding for this work.”***

3. We note that your Data Availability Statement is currently as follows: [All relevant data are within the manuscript and its Supporting Information files.]

Please confirm at this time whether or not your submission contains all raw data required to replicate the results of your study. Authors must share the “minimal data set” for their submission. PLOS defines the minimal data set to consist of the data required to replicate all study findings reported in the article, as well as related metadata and methods (<https://journals.plos.org/plosone/s/data-availability#loc-minimal-data-set-definition>).

For example, authors should submit the following data:

- The values behind the means, standard deviations and other measures reported;

- The values used to build graphs;

- The points extracted from images for analysis.

Authors do not need to submit their entire data set if only a portion of the data was used in the reported study.

If your submission does not contain these data, please either upload them as Supporting Information files or deposit them to a stable, public repository and provide us with the relevant URLs, DOIs, or accession numbers. For a list of recommended repositories, please see <https://journals.plos.org/plosone/s/recommended-repositories>.

If there are ethical or legal restrictions on sharing a de-identified data set, please explain them in detail (e.g., data contain potentially sensitive information, data are owned by a third-party organization, etc.) and who has imposed them (e.g., an ethics committee). Please also provide contact information for a data access committee, ethics committee, or other institutional body to which data requests may be sent. If data are owned by a third party, please indicate how others may request data access.

**Answer: *As requested, we hereby confirm that our submission contains all raw data required to replicate the results of this study.***

Additional Editor Comments

4. Please be more cautious with use of “childhood trauma”, while referring to the definition of childhood adversities. This requires caution throughout the manuscript. When using trauma I would recommend using the DSM-criteria of trauma exposure.

When referring to emotional abuse, physical abuse, sexual abuse and physical and emotional neglect I would recommend using “childhood maltreatment”.

**Answer: *Thank you for your comment. We revised the manuscript in order to avoid interchangeable use of terms regarding childhood adversity, maltreatment and trauma. For example (line 79, p.4), “Childhood trauma” was replaced by “Childhood adversity”. In addition, we decided to describe the nature of reported adverse or traumatic events by the included studies more explicitly within the Results section (objective 1, p 37-38). For example (line 227, p. 38):***

***“Only two studies assessed childhood trauma exposure based on PTSD qualifying stressors following DSM criteria.”***

***In general, this review addresses, as mentioned in the Introduction section, the exposure of traumatic and adverse events in relation to PTSD and CPTSD through the lens of both DSM and ICD diagnostic criteria which provide distinct diagnostic conceptual frameworks. For example, following current ICD-11 guidelines, childhood trauma exposure has been recently expanded by including different types of interpersonal trauma, in particular childhood neglect and emotional abuse, in addition to DSM criterion A events. We chose to mention this more explicitly in the Introduction section (line 105, p.5). Further, consistent with recent data (e.g., Frewel et al., 2019; Cloitre et al., 2019) and earlier conceptual research in relation to complex PTSD (e.g., Van der Kolk; Cloitre et al., 2009), we decided to use childhood trauma for the purpose of this review as a general term when referring to the exposure to traumatic and adverse events before the age of 18. We chose to clarify this within the Introduction (line 121, p. 6):***

***“For the purpose of this review, in line with previous conceptual research and current ICD-11 PTSD/CPTSD guidelines, the term childhood trauma is used to address the exposure of traumatic or adverse events before the age of 18 years.”***

**Review Comments to the Author**

5. Reviewer #1: This review is interesting and timely. The link between trauma in childhood, PTSD and cPTSD and chronic pain conditions is important and summarising the available evidence to draw clinical perspectives is welcome.

**Answer: *We thank the reviewer for this positive assessment.***

5.1 Overall, the review is well structured. However, the manuscript could be improved. In some sections, especially toward the Discussion, the grammar and syntax of sentences were really not clear; with some mistakes that can be avoided (e.g. Quality of studies of the included studies could just read as Quality of the included studies.)

**Answer: *We thank the reviewer for this insight. We revised and modified the manuscript in order to improve grammar and syntax of sentences. Additionally, we corrected the following mistake “Quality of studies of the included studies” into “Quality of the included studies” (line 200, p. 35).***

5.2. It was surprising to see the authors inspecting PROSPERO to make sure there was no other ongoing review on the topic, but why did not they register this systematic review in PROSPERO?

**Answer: *We understand this observation. As this research study was conducted as part of a dissertation project, it was initially uncertain whether a second reviewer would be available for data selection and extraction. When this was confirmed, the first author already started study selection and therefore the protocol did no longer meet eligibility criteria for registration with PROSPERO. We decided not to mention this detail within the manuscript.***

5.3. I was surprised by the relatively low number of records generated by the search. And when using their search strategy in Pubmed only, I received 13,646 results. Of course, not all are relevant, but the discrepancy is massive. Could the authors explain how they ended up with only 297 records from their search? Did the authors used tools like Covidence? Maybe the authors will want to update their search while revising the manuscript?

**Answer: *In fact, parenthesis “( )” were used for the search syntax. In order to promote data replicability, we added these within Table 1 of the manuscript (line 146, p. 7). Given the fact that this modification didn’t impact search results, we decided not to update the search.***

***We did not use Covidence. Other software, namely Excel and Zotero, were used to streamline the process of conducting this systematic review.***

5.4. What was the authors strategy to estimate the certainty of evidence if they did not use GRADE? The Joanna Briggs Institute (JBI) Critical Appraisal Checklist tool is dedicated to systematic reviews and research syntheses, not for empirical studies.

**Answer: *We thank the reviewer for this comment. We did not use the JBI critical appraisal tool for systematic reviews. Instead, the methodological quality of each included study was assessed separately by using the corresponding design-specific critical appraisal checklist provided by JBI, including those for case-control studies, analytical cross-sectional studies, cohort studies and quasi-experimental studies (available from*** [***https://jbi.global/critical-appraisal-tools***](https://jbi.global/critical-appraisal-tools)***). We chose to address this information more explicitly in the Methods section (line 167, p. 11).***

***In addition, based on previous systematic reviews using JBI critical appraisal checklist tools (e.g., Dijkshoorn et al., 2021; Whittaker et al., 2022), we decided to classify estimated levels of evidence as follows:***

- ***High quality studies: scores > 70%***
- ***Moderate quality studies: scores between 50% and 70%***
- ***Low quality studies: scores < 50%***

***This information was added in the Methods section (line 174, p.11). In order to enhance coherence within the manuscript, total quality scores were adjusted so to reference percentages instead of total scores in Table 5 (line 208, p 35).***

5.5. The description of the informations extracted from the publications is sometimes difficult to follow. I am sure the authors could do better to summarise the results, and could also include intermediate conclusions to help the reader to get the take home message after a long succession of summaries...

**Answer: *We thank the reviewer for this suggestion. We revised the Results section (p. 36 – 44) in order to improve summary coherence and readability of extracted information.***

5.6. Study objective 3 confused me. Why not integrating this section to the Discussion where it would be more appropriate?

**Answer: *We thank the reviewer for this observation. Study objective 3 was articulated in response to the current lack of available translational and clinical research highlighted in the Introduction section. Resulting findings also add to existing data, as this, considering the literature, has not been systematically reviewed by previous research. We chose to reframe the third study objective (line 132, p. 6) to better distinguish results from study perspectives that are addressed in the Discussion section.***

5.7. The discussion could end on a short conclusion section to summarise the overall take home message of the manuscript, as well as clinical and research perspectives.

**Answer*: We thank the reviewer for this suggestion. A short conclusion was added to highlight main findings of the manuscript as well as recommendations for future research and clinical practice (line 546, p. 50)***

6. Reviewer #2: I applaud the efforts of the author to bring light to the issue of a lack of rigorous scientific research in the area of childhood abuse as associated with trauma related conditions such as chronic pain and PTSD/CPTSD. I find the paper well written with the methodology easy to follow and comprehend. The results do not extend beyond the data and are appropriate. I only have a few suggestions to perhaps add to the limitations section.

**Answer: *We thank the reviewer for this positive assessment.***

6.1. Were the studies selected using appropriate assessment methods for BOTH chronic pain and PTSD? If so, it should be highlighted but, if not, it is definitely a limitation for future research to address.

**Answer: *Overall, the selected studies used appropriate and validated measurement for childhood trauma, PTSD/CPTSD and chronic pain which included a variety of self-reported as well as physician-based assessment. We added this information in the Methodological considerations section (line 528, p. 49) with some additional comments to consider in relation to PTSD and childhood trauma assessment.***

6.2. Did any of the studies selected look at PTSD symptom cluster in association with childhood trauma. If so, it should be highlighted but, if not, it is another limitation for future research to address.

**Answer: *Only one study selected in this manuscript reported PTSD cluster symptoms in a community sample of women with FM versus healthy controls (Ciccone et al., 2005). We chose to highlight this information in the Results section (line 252, p. 39).***

6.3. What about the PTSD symptoms of dissociation or depression? Research I've done in this area indicated the it is "... among women with childhood sexual or physical abuse, depression was the strongest predictor of somatic symptoms, whereas only dissociation predicted somatic symptoms among women without childhood sexual or physical abuse. Understanding the psychological and biological mechanisms that link PTSD comorbid depression or dissociation to physical health symptoms may aid development of individualized treatments for the physical and psychological consequences of trauma. In this context, a history of childhood trauma should also be taken into account." I recall seeing depression discussed in this review but dissociation should also be discussed or at least mentioned in the limitations section to be a part of assessment in future research.

**Answer: *We thank the author for this insight. We decided to add this information in the Discussion section (line 459, p. 47) to highlight the importance of considering trauma childhood versus adulthood trauma exposure in relation to differential pathways in PTSD-related comorbidity and chronic pain.***
